# 163. Novel Factors Predicting Mortality in Allogeneic Hematopoietic Cell Transplantation Recipients with Viral Upper Respiratory Tract Infection

**DOI:** 10.1093/ofid/ofae631.049

**Published:** 2025-01-29

**Authors:** Chikara Ogimi, Hu Xie, Alpana waghmare, Keith R Jerome, Wendy M Leisenring, Masumi Ueda, Paul A Carpenter, Janet A Englund, Michael J Boeckh

**Affiliations:** National Center for Child Health and Development, Setagaya-ku, Tokyo, Japan; Fred Hutchinson Cancer Center, Seattle, Washington; Fred Hutchinson Cancer Center; Seattle Children's Hospital, Seattle, Washington; Fred Hutchinson Cancer Center, Seattle, Washington; Fred Hutchinson Cancer Center, Seattle, Washington; University of Washington/Fred Hutchinson Cancer Research Center, Seattle, Washington; Fred Hutchinson Cancer Research Center; University of Washington, Seattle, Washington; Seattle Children’s Hospital, Seattle, Washington; Fred Hutchinson Cancer Center, Seattle, Washington

## Abstract

**Background:**

We previously identified novel risk factors for progression to lower respiratory tract disease (LRTD) among allogeneic hematopoietic cell transplant (HCT) recipients presenting with upper respiratory tract infection (URTI) with 12 viruses in the PCR era and proposed a simple prediction model by a number of risk factors (BMT Ogimi C, et al. Bone Marrow Transplant 2022; 57(4):649-657). We aimed to investigate whether the presence of these risk factors at the time of URTI diagnosis also predicts overall mortality and pulmonary death.

**Figure 1.**
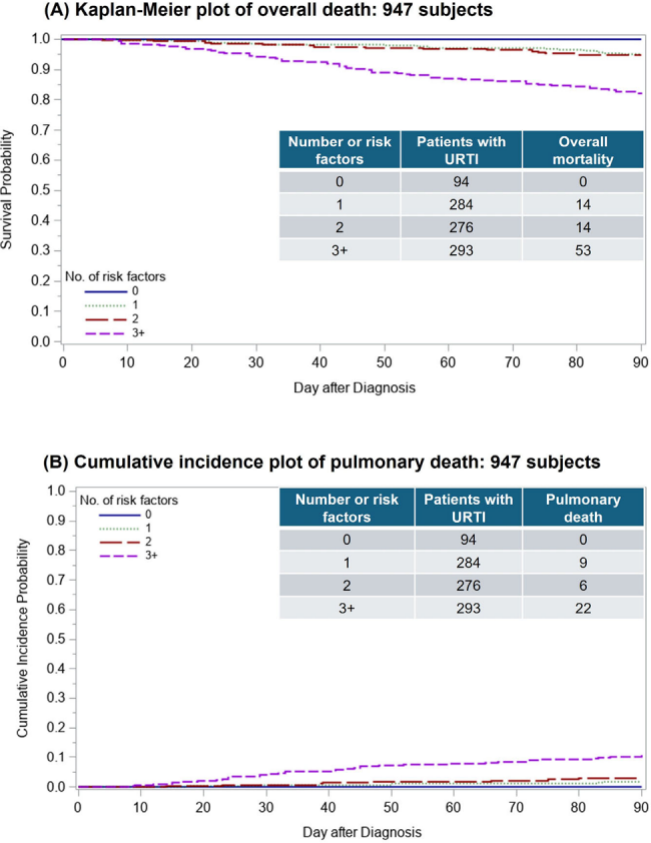
Kaplan-Meier plot of overall death and cumulative incidence plot of pulmonary death by day 90 among patients with any viral URTI (N=947)

**Methods:**

We retrospectively analyzed patients with the first respiratory virus detected by multiplex PCR after allogeneic HCT (4/2008-9/2018). We created Kaplan-Meier plots for overall mortality and cumulative incidence plots for pulmonary death within 90 days among patients presenting with URTI by a single virus. Candidate risk factors were age >=40 years, a history of multiple HCT, early timing post-HCT (< =30 days), albumin < =3 g/dL, monocytopenia (< =100 cells/µL), highest glucose value >200 mg/dl, and systemic steroid use within 14 days.

**Figure 2.**
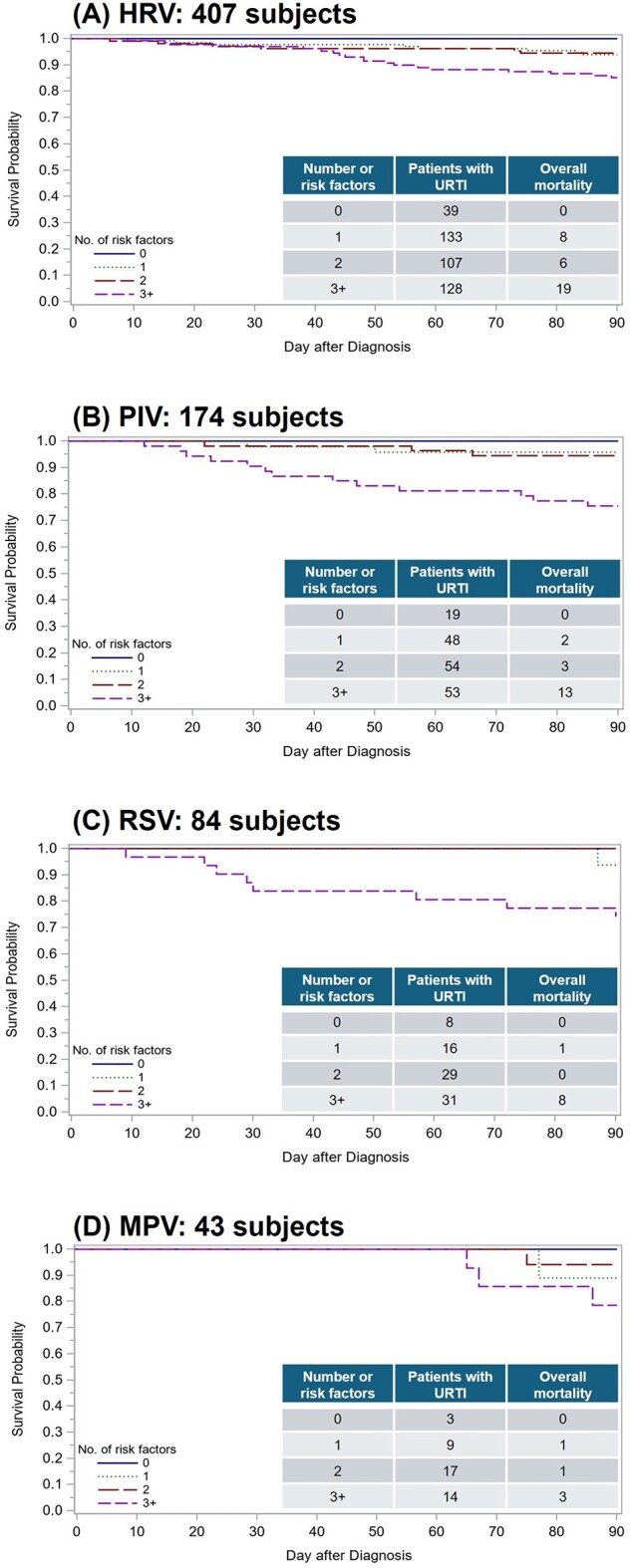
Kaplan-Meier plot of overall death by day 90 among patients with viral URTI

**Results:**

A total of 947 patients (199 children and 748 adults) presented with URTI only as follows: 407 human rhinoviruses (HRV), 174 parainfluenza viruses (PIV) 1–4, 140 common cold coronaviruses, 84 respiratory syncytial virus (RSV), 58 influenza A/B, 43 human metapneumovirus (MPV), and 41 adenoviruses. Among these, 81 (18%) died with 44 pulmonary deaths within 90 days from URTI. No patients with any viral URTI without risk factors died **(Figure 1)**. Patients with 3 or more risk factors appear to have high overall mortality, and pulmonary death accounted for more than half of cases as a cause of death **(Figure 1)**. These trends were seen across viruses evaluated (**Figure 2)**.

**Conclusion:**

Established risk factors for progression from viral URTI to LRTD also predict overall mortality and pulmonary death. Patients without any risk factors appeared to be completely protected from death, while patients with >3 risk factors were at the highest risk for death, suggesting an intervention opportunity and close monitoring. These trends were observed across several viruses and future studies are needed to validate our findings in different cohorts.

**Disclosures:**

**Chikara Ogimi, MD, PhD**, AstraZeneca: Honoraria|bioMerieux Japan Ltd.: Honoraria|ELSEVIER: Honoraria|KYORIN: Honoraria|Miyarisan: Honoraria|MSD: Honoraria|NOVARTIS: Honoraria|Pfizer: Honoraria **Alpana waghmare, MD**, Allovir: Grant/Research Support|Ansun Biopharma: Grant/Research Support|GlaxoKlineSmith: Advisor/Consultant|GlaxoKlineSmith: Grant/Research Support|Pfizer: Grant/Research Support|Vir: Advisor/Consultant **Janet A. Englund, MD**, Abbvie: Advisor/Consultant|AstraZeneca: Advisor/Consultant|AstraZeneca: Grant/Research Support|GlaxoSmithKline: Advisor/Consultant|GlaxoSmithKline: Grant/Research Support|Meissa Vaccines: Advisor/Consultant|Merck: Advisor/Consultant|Pfizer: Board Member|Pfizer: Grant/Research Support|Pfizer: Speaker at meeting|SanofiPasteur: Advisor/Consultant|Shinogi: Advisor/Consultant **Michael J. Boeckh, MD PhD**, Allovir: Advisor/Consultant|Allovir: Grant/Research Support|AstraZeneca: Advisor/Consultant|AstraZeneca: Grant/Research Support|Merck: Advisor/Consultant|Merck: Grant/Research Support|Moderna: Advisor/Consultant|Moderna: Grant/Research Support|Symbio: Advisor/Consultant

